# A *CLIP* on the Ear: Spitz Melanocytoma Harbouring a *CLIP2-BRAF* Gene Fusion

**DOI:** 10.1155/crdm/1460562

**Published:** 2026-01-27

**Authors:** Karwan A. Moutasim, Mohammed Atweh, Jeffery M. Theaker

**Affiliations:** ^1^ University Hospital Southampton NHS Foundation Trust, Southampton, UK, nhs.uk; ^2^ Hampshire Hospitals NHS Foundation Trust, Winchester, UK, hampshirehospitals.nhs.uk

## Abstract

Spitzoid lesions represent one of the most challenging areas in melanocytic pathology. Many such lesions are characterised by key gene alterations including *ALK, ROS* and *NTRK* fusions. *BRAF* mutations are generally considered incompatible with the diagnosis of Spitz tumours. Here, we present the case of a spitzoid melanocytoma harbouring a rare *BRAF* gene fusion. A brief overview of the literature is also touched upon.

## 1. Case

Spitzoid lesions represent one of the most challenging areas in melanocytic pathology. The original case series published by Sophie Spitz highlighted a series of paediatric Spitz lesions [1], the majority of which were likely Spitz naevi. It has now been over 75 years since her landmark contribution to the literature, and our understanding of these histologically heterogeneous lesions has been substantially improved by the advent of molecular and genomic techniques that the histopathologist in categorising and ultimately providing a clinically meaningful diagnosis.

It is now accepted that Spitzoid melanocytic tumours represent a spectrum ranging from benign Spitz naevi at one end and Spitzoid melanomas at the other with the intermediate category of Spitz melanocytoma (atypical Spitz tumour) also increasingly recognised. The majority of Spitz lesions are driven by fusions in receptor tyrosine kinase genes (e.g., *ALK, ROS1, NTRK1-3* and *RET*) [2, 3]. Less commonly, serine threonine gene fusions may also be seen (e.g., *BRAF*) [4].

A 37‐year‐old male presented with a polypoidal lesion in the right postauricular area clinically thought to be neurofibroma. Macroscopic description showed a skin ellipse 13 × 12 × 3 mm with a centrally attached polypoidal nodule, 18 × 15 × 9 mm. Histological examination showed a predominantly intradermal melanocytic proliferation with a symmetrical, wedge‐shaped silhouette (Figure 1(a)). Epithelioid melanocytes with voluminous cytoplasm and prominent nucleoli were seen (Figure 1(b)). A prominent sclerotic stroma was noted (Figure 1(c)). Occasional multinucleated cells were present. Mitotic activity was rare and appeared confined to the superficial aspect of the lesion.

Figure FIGURE 1(a) A polypoidal melanocytic lesion with a fairly symmetrical outline. (b) Epithelioid melanocytes with voluminous cytoplasm, vesicular nuclei and identifiable nucleoli. (c) Fibrous stroma harbouring scattered epithelioid melanocytes, with occasional multinucleated cells.

**Figure:**
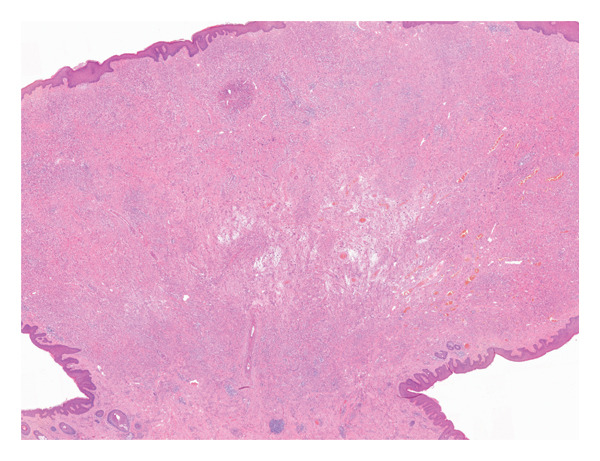
(a)

**Figure:**
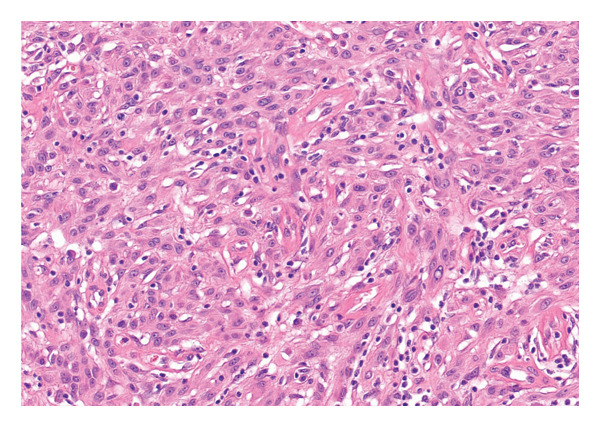
(b)

**Figure:**
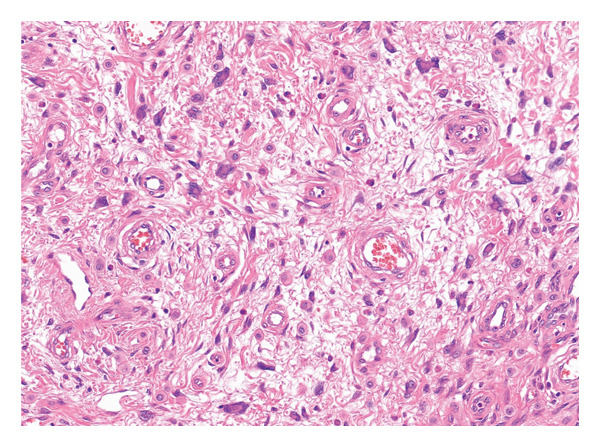
(c)

*ALK* and *ROS1* immunohistochemistry were negative, as was PRAME. The MIB1 proliferation index was low. A retained, checkerboard pattern p16 pattern was seen. An initial provisional diagnosis of atypical Spitz tumour (Spitz melanocytoma) was issued, pending comprehensive targeted next generation sequencing (NGS) utilising the ArcherFusion panel.

NGS results showed a *CLIP2-BRAF* gene fusion (Figure 2). BRAF fusions in general are rare in Spitz tumours and are found in less than 5% of these lesions [4]. A recent case report had also interestingly highlighted similar morphological features, including a sclerotic stroma [5].

**Figure FIGURE 2:**
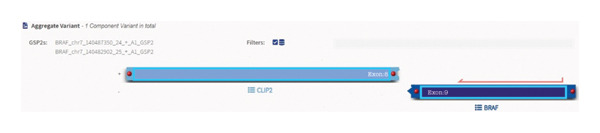
Schematic of *CLIP2-BRAF* gene fusion.

A limited re‐excision together with discussion at multidisciplinary meeting (MDT) and appropriate surveillance was performed. Sentinel lymph node biopsy was not performed. To date, the patient remains well with no evidence of recurrent or metastatic disease.

Botton and colleagues recently performed a systematic review of BRAF fusions in melanocytic tumours, identifying 100 such cases [4]. These had varying morphological patterns ranging from banal naevi to frank melanomas. Spitzoid features were seen in more than half of the cases reviewed. Here, we present a case of Spitz melanocytoma with a rare *CLIP2-BRAF* gene fusion. The key learning point is to interpret this correctly as a *BRAF* gene fusion as the majority of these lesions behave in an indolent manner, with a rare propensity to progression to melanoma. *BRAF V600E* gene mutations are rare in Spitz melanocytic lesions and should be interpreted with caution, as the majority of these may represent frankly malignant melanomas [6].

Further studies are required to elucidate the exact role of *BRAF* gene fusions and mutations in the spectrum of Spitzoid melanocytic lesions.

## Author Contributions

K.A.M. wrote the manuscript. M.A. and J.M.T. also contributed to writing the manuscript.

## Funding

No funding was received for this manuscript.

## Consent

No written consent has been obtained from the patient as there are no patient identifiable data included in this case report.

## Conflicts of Interest

The authors declare no conflicts of interest.
